# Biofabrication of *Acer palmatum*-Mediated Multifunctional CuO Nanoparticles for Dye Removal, Antibacterial–Antifungal Activity, and Molecular Docking

**DOI:** 10.1021/acsomega.3c03591

**Published:** 2023-09-25

**Authors:** Cansu Sazak, Azade Attar, Alper Yilmaz, Melda Altikatoglu Yapaoz

**Affiliations:** † Faculty of Science and Letters, Department of Chemistry, Davutpasa Campus, Yildiz Technical University, Istanbul 34220, Turkey; ‡ Faculty of Chemical & Metallurgical Engineering, Department of Bioengineering, Davutpasa Campus, Yildiz Technical University, Istanbul 34220, Turkey

## Abstract

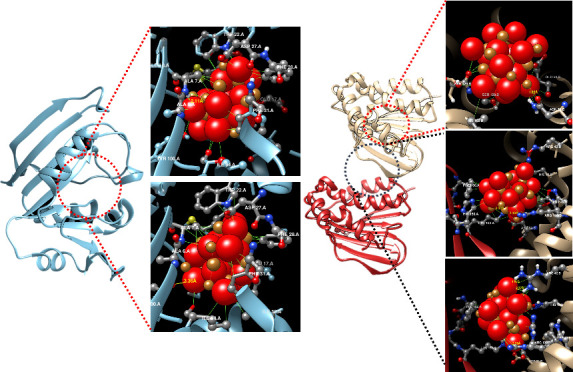

Copper oxide nanoparticles (CuONPs) are used in many fields from electronics to medicine due to their multifunctionality, and therefore, their production with environmentally friendly methods is a current issue. In this study, biofabricated CuONPs were obtained by using the leaf extract of *Acer palmatum* plant originating from the Far East to enlighten the characteristics of the novel nanoparticles differentiating from those existing in the literature. Multifunctional nature of the CuONPs was evaluated by the antibacterial, antifungal, and decolorative applications and also by performing molecular docking analysis. The fabricated CuONPs were characterized using ultraviolet-visible spectroscopy (UV–vis), Fourier-transform infrared spectroscopy (FT-IR), scanning electron microscopy (SEM), and dynamic light scattering (DLS). The absorbance seen at 270 nm in the SPR band obtained by UV–vis spectroscopy proved the presence of CuONPs, while the 602, 560, and 540 cm^–1^ vibrations obtained in the FT-IR spectroscopy indicated the same result. SEM images proved that the nanoparticles were in spherical form with sizes ranging from 140 to 225 nm. The result of DLS analysis showed that the average particle size was 229 nm in diameter, and CuONPs had monodisperse systems (polydispersity index, 0.184). The dye removal potency of CuONPs was also investigated by using remazol brilliant blue R (RBBR) and napthol blue black (NBB). Decolorizations (74 and 86%) of RBBR and NBB were obtained in 90 min at 50 °C, respectively. The strong antibacterial properties of the synthesized CuONPs were observed on both Gram (−) and Gram (+) bacterial strains by disk diffusion and optical analyses, and their antifungal activity was close to that of Amphotericin B, which was applied as a positive control. Molecular docking analysis was performed with *Escherichia coli* dihydrofolate reductase and *Staphylococcus aureus* DNA Gyrase B to analyze the antibacterial mechanisms of CuONP and observed that they exhibit good interactions with their targets with binding energies of −12.562 and −8.797 kcal/mol, respectively. Our findings suggested that CuONPs are crucial in the mechanisms of folate metabolism and DNA replication associated with bacterial proliferation. This work will provide significant guidance for the biofabrication of CuONPs and their medical and industrial applications.

## 1 Introduction

The scientific community has made extensive efforts to develop suitable synthetic techniques to produce nanoparticles due to the physicochemical properties of nanoparticles and their applications in many fields. However, in chemical and physical methods, high radiation and high concentration reducing and stabilizing agents that are harmful to the environment and human health are used. Biological nanofabrication of nanoparticles, on the other hand, is an environmentally friendly, one-step bioreduction method and uses less energy.^1^ Various physiochemical approaches for the synthesis of metal nanoparticles have been restricted due to environmental pollution caused by heavy metals. Organisms have evolved to withstand environments with high concentrations of metals. These organisms can alter the chemical makeup of toxic metals by reducing their toxicity or making them nontoxic. Because of these properties of organisms, bacteria, fungi, yeast, and plants have been shown as new sources with significant potential for synthesizing nanoparticles. Thus, the synthesis of nanoparticles by biological means has become a new trend due to its advantages such as nontoxicity, reproducibility in production, easy scalability, and well-defined morphology.^2^ The synthesis using bio-organisms is compatible with the principles of green chemistry. Green chemistry refers to the synthesis of nanoparticles or nanomaterials with the help of various biotechnological means using biological pathways, such as microorganisms and plants, or their byproducts, such as proteins and lipids. In the green synthesis of nanoparticles, environmentally friendly, nontoxic, safe reagents are used, eliminating the use of expensive chemicals, less energy is consumed, and environmentally harmless products and byproducts are produced. Nanoparticles synthesized using biological techniques or green technology are far superior to those produced by physical and chemical methods in several aspects, such as greater stability and convenient dimensions, since they are synthesized using a one-step procedure.^3^ Since plant and microorganism components act as stabilizing agents, no other stabilizing agents are needed in biological synthesis.^4^ Among the existing green synthesis methods for metal/metal oxide nanoparticles, the use of plant extracts is a simpler and easier process to produce nanoparticles on a large scale than bacterial or fungal mediated synthesis. Green synthesis products based on biological precursors depend on various reaction parameters such as solvent, temperature, pressure, and pH conditions. For the synthesis of metal/metal oxide nanoparticles, plant biodiversity has been widely addressed due to phytochemicals such as aldehydes, ketones, flavones, carboxylic acids, terpenoids, phenols, ascorbic acids, and amides found in various plant extracts, especially in leaves.^5^ These components have the ability to reduce metal salts to metal nanoparticles. Such nanomaterials are being investigated for use in fields such as biomedical diagnostics, antimicrobials, molecular sensing, optical imaging, and labeling of biological systems.^6^

Easy and environmentally friendly production of metal oxide nanoparticles is increasing day by day due to their specific surface areas and high densities, unique physical and chemical properties, and applications in medicine, optics, biotechnology, and photocatalysis.^7−9^ Compared to other metals such as silver, platinum, and gold, copper, which is easily available and known to be nontoxic to mammals, is frequently used in antibacterial, electrical, and optical applications due to its low cost and favorable oxidation properties.^10^ Among other transition metals, copper oxide nanoparticles (CuONPs) have attracted more attention due to their effectiveness in catalysis, superconductivity, batteries, biomedical field, biosensor, energy storage devices, heat transfer applications, and antimicrobial activity.^11^ The properties of CuONPs are very important for their applications in various fields such as biomedical research, the use of which is most dominant, and the properties of the nanoparticles depend on the chosen synthesis method. The most important feature of nanoparticles is their nanoparticle size, which can be controlled during synthesis. This is because it allows special modeling of optical, catalytic, electrical, and biological properties. The chosen synthesis method is an important parameter to control the size, surface properties, application areas, and biological properties of nanoparticles.^12^ Copper-based nanostructures are promising materials due to some unique properties over other metallic nanoparticles. Copper is highly reactive and can perform a variety of catalytic reactions involving one- or two-electron paths due to its wide range of oxidation degrees (Cu^0^, Cu^I^, Cu^II^, and Cu^III^). These properties can be used to design and build third-generation sensors for physiologically relevant electro-active analytes such as ascorbic acid, dopamine, glucose, and l-cysteine.^13^ Because copper has antifungal, antibacterial, and anti-inflammatory properties, copper-based nanomaterials are suitable candidates for the design of microbe-resistant medical devices, bandages, and ointments.^14^

In this study, which was carried out in light of the information summarized above, biological nanofabrication of CuONPs from *Acer palmatum* plant was performed for the first time in the literature. The plant is available in the Marmara region of Turkey, and the leaves were collected from Istanbul. *A. palmatum* (Japanese Maple), which belongs to the *Sapindaceae* family, grows in many parts of the world, especially in China and Japan. *A. palmatum* leaf, which contains flavonoids such as anthocyanin with superior bioactivity, has various phytochemicals, and is known to have antioxidant, antitumor, and anti-inflammatory properties, was used for the synthesis of CuONPs in this study.^15−17^ . The aim of this study was to evaluate the multifunctional nature of the biofabricated CuONPs by employing antimicrobial and water clean up processes and by in silico analysis. The obtained CuONPs were characterized; their antibacterial and antifungal properties were investigated, and molecular docking analysis was performed for the in silico antibacterial evaluation of the obtained CuONPs as a first report. In addition, the dye removal potential was measured employing napthol blue black and remazol brillant blue R at various temperatures to evaluate the color removal activity of the biologically fabricated CuONPs.

## 2 Materials and Methods

### 2.1 Biological Nanofabrication of CuONPs

*A. palmatum* leaves collected from the Sarıyer/Istanbul region were dried in an oven at 50 °C for 18 h. Five grams of the dry plant was taken and extracted with 50 mL of distilled water for 10 min at 60 °C. The extract, which was filtered with filter paper when it came to room temperature, was completed to 100 mL with distilled water, and the pH was adjusted to 5.0. The plant extract (50 mL) was taken, 1 mL of 0.1 M Cu(SO_4_)·5H_2_O solution was added to it, and it was left to mix for synthesis in a 60 °C water bath for 2 h. After three washing processes, it was centrifuged for 5 min and dried at 50 °C overnight.

### 2.2 Optimization of CuONP Fabrication

In the optimization study, CuONPs were synthesized using the *A. palmatum* leaf extract at different synthesis rates. The ratios chosen for the optimization study (extract:Cu(SO_4_)·5H_2_O solution) are 5:1, 10:1, 20:1, 30:1, 40:1, and 50:1 (v/v) at pH 5.0. The optimum synthesis rate was determined by comparing the absorbance values of CuONPs obtained at the synthesis rates by ultraviolet-visible (UV–vis) spectroscopy. Also, CuONP synthesis was performed for each pH value by adjusting the leaf extract to pH 3.0, 5.0, 7.0, 8.0, 9.0, and 10.0 using a concentration rate of 50:1 (extract:Cu(SO_4_)·5H_2_O solution, v/v). The synthesis was also applied at pH 4.7, the natural pH of the plant extract.

### 2.3 Characterization of CuONPs

UV–vis, Fourier-transform infrared spectroscopy (FT-IR), scanning electron microscopy (SEM), and DLS methods were used for the characterization of CuONPs synthesized from *A. palmatum* leaf extract. The spectroscopic analysis was carried out at room temperature in a cuvette with a path length of 10 mm using a Shimadzu UV2400. At 200–800 nm, analyses were performed in relation to response time. With the aid of a PerkinElmer 1600 instrument, CuONPs were examined using FT-IR to reveal details about the binding properties of the obtained nanoparticles. The plant extract was mixed with potassium bromide (KBr) at a ratio of 1:10 (v/v), and the resulting particle was observed between 400 and 4000 cm^–1^ of the wavenumber. A Hitachi S5500 was used in the SEM tests to determine the CuONP shape. To make SEM samples, CuONPs were first applied to carbon-coated grids and then washed twice with ultrapure water. The average particle size and polydispersity index (PDI) value of the synthesized CuONPs were determined by performing DLS analysis with a Zetasier Malvern Nano ZS device.

### 2.4 Antibacterial Activity of CuONPs

Antibacterial activities of CuONPs were determined by a disk diffusion test using Gram (−) *Escherichia coli* and Gram (+) *Staphylococcus aureus* bacterial strains. Both strains were incubated for 24 h at 37 °C in a nutrient broth medium. Growing colonies were diluted with saline to 10^8^ CFU/mL according to the McFarland 0.5 turbidity standard. Twenty microliters of the prepared bacterial suspensions was taken and inoculated on nutrient agar. CuONP solution (1 mg/mL) was added to the wells of approximately 5 mm in diameter on the medium and incubated at 37 °C for 24 h. Zone diameters (mm) formed after incubation were measured and evaluated. Streptomycin was used as a positive control, and distilled water was used as a negative control. Also, optical analysis was applied by incubating the strains overnight at 37 °C in nutrient broth. One milliliter of broth without bacterial cells (negative control) and 1 mL of CuONP solution were mixed with 2 mL of broth including bacterial cells. This mixture was incubated at room temperature for 24 h in UV cuvettes, and OD_600_ data was collected at 1, 2, 3, 4, 6, 8, and 24 h. All experiments were made in triplicate.

### 2.5 Antifungal Activity of CuONPs

Antifungal susceptibility of synthesized CuONPs was evaluated by the agar diffusion test against the stock culture of yeast *Aspergillus niger*. Fungal cells were cultivated in a potato dextrose agar (PDA) medium for pretest growth. The PDA medium was also used in the disk diffusion method to examine the antifungal activity of CuONPs. According to McFarland 0.5 turbidity, standard microorganisms were adjusted to 10^8^ CFU/mL. Twenty microliters of activated *A. niger* was inoculated in the medium and was incubated at 37 °C for 24 h. Amphotericin B, an antifungal antibiotic, was used as the positive control, and distilled water was used as the negative control. The zones formed at the end of 24 h were measured and compared with the zones of Amphotericin B. Also, quantification was processed by incubating the strains overnight at 37 °C in potato dextrose broth. One milliliter of broth without fungal culture (negative control) and 1 mL of CuONP solution were mixed with 2 mL of broth including fungal cells. This mixture was incubated at room temperature for 24 h in UV cuvettes, and OD_600_ data was collected at 1, 2, 3, 4, 6, 8, and 24 h. All experiments were performed in triplicate.

### 2.6 Determination of Dye Removal Efficiency of CuONPs

Naphthol blue black (NBB) and remazol brilliant blue (R) (RBBR) were applied in the dye removal determination studies of the fabricated CuONPs. The decolorization effect of the CuONPs was determined by reacting 2 mg/mL CuONPs with 10 mg/L NBB and RBBR at pH 5.0. The experiments were conducted at two different temperatures, 25 and 50 °C. The reaction depends on the removal of color, and the degradation capacity of CuONPs was measured for 90 min to evaluate the impact of time on the dye degradation process. The percentage of degradation was quantified spectrophotometrically employing a Shimadzu Pharmaspec UV-1700. All experiments were made in triplicate. The following formula was used to calculate the decrease in the absorbance at the specific λ_max_ of dyes:
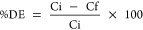


where DE is the dye removal efficiency, Ci is the absorbance of the dye, and Cf is the absorbance of the dye upon contact with the CuONPs.

### 2.7 Molecular Docking of CuONPs

The molecular docking of CuONPs was performed by using *E. coli* dihydrofolate reductase (DHFR, PDB entry 5CC9) and *S. aureus* DNA Gyrase B (GyrB, PDB entry 3U2D) to assess CuONP antibacterial molecular mechanisms. The structure of monoclinic CuONPs was constructed, and geometry optimization via energy minimization with a universal force field (UFF) was applied in Avogadro molecular editor software (version 1.2).^18^ The local molecular docking analysis was performed in AutoDock Vina (version 1.2.0), and the blind (also called as global docking) was performed via an HDOCK web server.^19,20^ All structure and binding analyses were carried out by using UCSF Chimera (version 1.16).^21^

## 3 Results and Discussion

CuONPs were obtained via *A. palmatum* leaf extract by biofabrication. Synthesis by a one-step bioreduction reaction is fast, easy to implement, environmentally friendly, and cost-effective.

### 3.1 UV–vis Analyses of CuONPs

**Figure 1:**
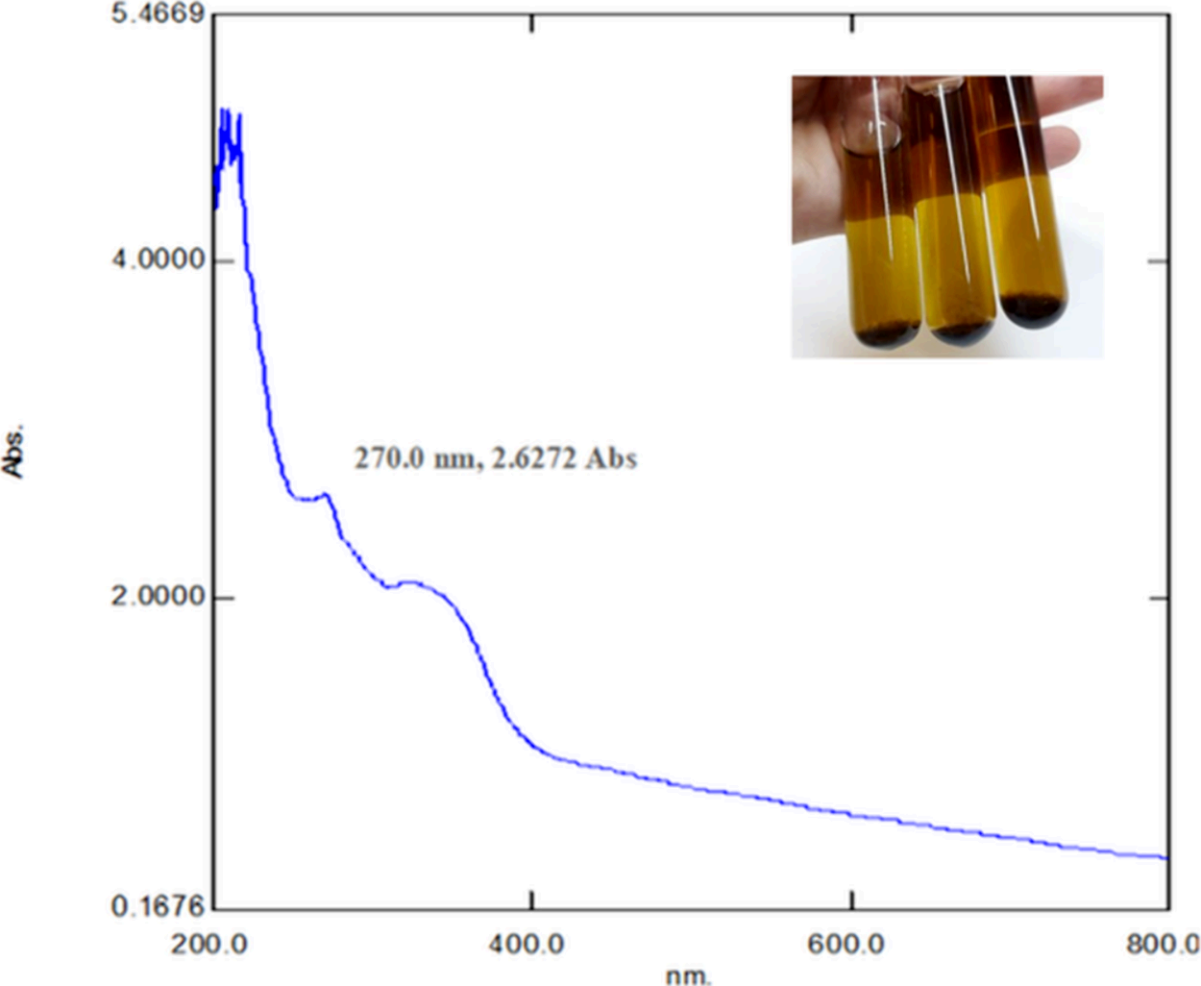
Absorption spectra of CuONPs synthesized via *A. palmatum*.

CuONPs obtained from *A. palmatum* leaf extract were analyzed by UV–vis spectroscopy in the wavelength range of 200–800 nm. It was observed that the maximum absorption band at 270 nm indicates CuONPs obtained via *A. palmatum* leaf extract (Figure 1). The dark precipitate seen in the test tubes also shows the morphologically detectable nanoparticle synthesis step. Similar results are found in the literature, CuONPs obtained with *Calotropis procera* leaf extract have absorbance with a sharp peak at 285 nm^22^, and the surface plasmon resonance of CuONPs synthesized using prickly pear leaf extract showed a broad band at 292 nm.^23^ The absorption peak at 270 nm strongly supports the purity and fine formation of CuONPs via *A. palmatum*. Therefore, the results confirmed that the nanoparticles obtained at the end of biological nanofabrication were pure copper oxide nanoparticles.

### 3.2 Optimization Studies

#### 3.2.1 Optimization of CuONPs According to Synthesis Rates

**Figure 2:**
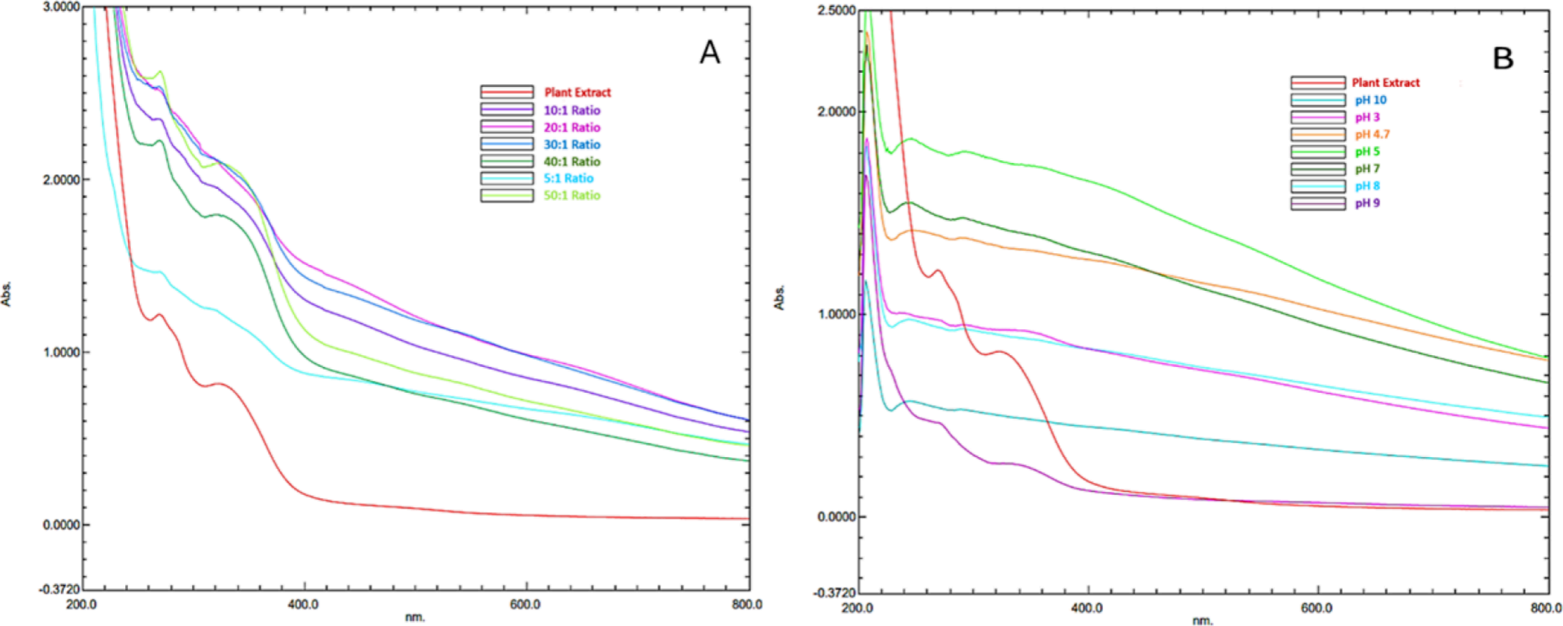
Absorption spectra of CuONPs obtained by using different (A) synthesis rates and (B) pH.

It was reported in the literature that smaller nanoparticles are responsible for the blue shift in the absorption spectrum, whereas bigger nanoparticles are responsible for the red shift.^24^ In this study, we tested different ratios of synthesis components to perform biofabrication in the most efficient way and to optimize CuONP synthesis. Surface plasmon resonance of CuONPs was measured after mixing *A. palmatum* plant extract:Cu(SO_4_)·5H_2_O solution at 5:1, 10:1, 20:1, 30:1, 40:1, and 50:1 (v/v) ratios. The rate with the highest absorbance value was accepted as the optimum synthesis rate in the study. Figure 2A shows the UV–vis spectrum of CuONPs obtained with different ratios of *A. palmatum* leaf extract and copper solution between 200 and 800 nm. The synthesis ratio with the highest absorbance value was observed as 50:1 (plant extract:copper solution, v/v) at pH 5.0, and this value was chosen as the optimum synthesis ratio. The remaining experiments were conducted using this ratio. In metal nanoparticle synthesis, ambient pH and concentration are two important parameters that affect the nanoparticle structure. The plant extract concentration is a crucial parameter to reduce and stabilize the nanoparticles in the production process.^25^ It is also important for improving the size, shape, and morphology. The spherical forms do not change as the concentration of the plant extract rises, but the sizes do. In other words, the nanoparticle size changes as the concentration of the phytoconstituents increases.

#### 3.2.2 Optimization of CuONPs According to pH

The pH has an important effect on the biofabrication of nanoparticles because the H^+^ ion concentration of the medium affects the size of the synthesized particle. It is possible to change the size of the product to be obtained by simply changing the pH. It is known that small-sized nanoparticles are obtained when the pH of the reaction medium is acidic, and large-sized nanoparticles are obtained when it is basic. In other words, the size of synthesized nanoparticles increases with the increase in the pH of the reaction mixture.^26^ Therefore, pH is one of the most important environmental parameters in nanoparticle synthesis studies. In this study, CuONPs were synthesized using plant extract at different pH values, and the absorbances were measured by UV–vis spectroscopy. The pH value with the highest absorbance was accepted as the optimum pH value of the plant extract used to synthesize CuONPs. Figure 2B shows the UV–vis result of CuONPs obtained with *A. palmatum* leaf extract at different pH values, measured between 200 and 800 nm. The pH value with the highest absorbance was observed as pH 5.0, and this value was chosen as the optimum pH. The remaining experiments were conducted at pH 5.0.

### 3.3 FT-IR Analyses of CuONPs

**Figure 3:**
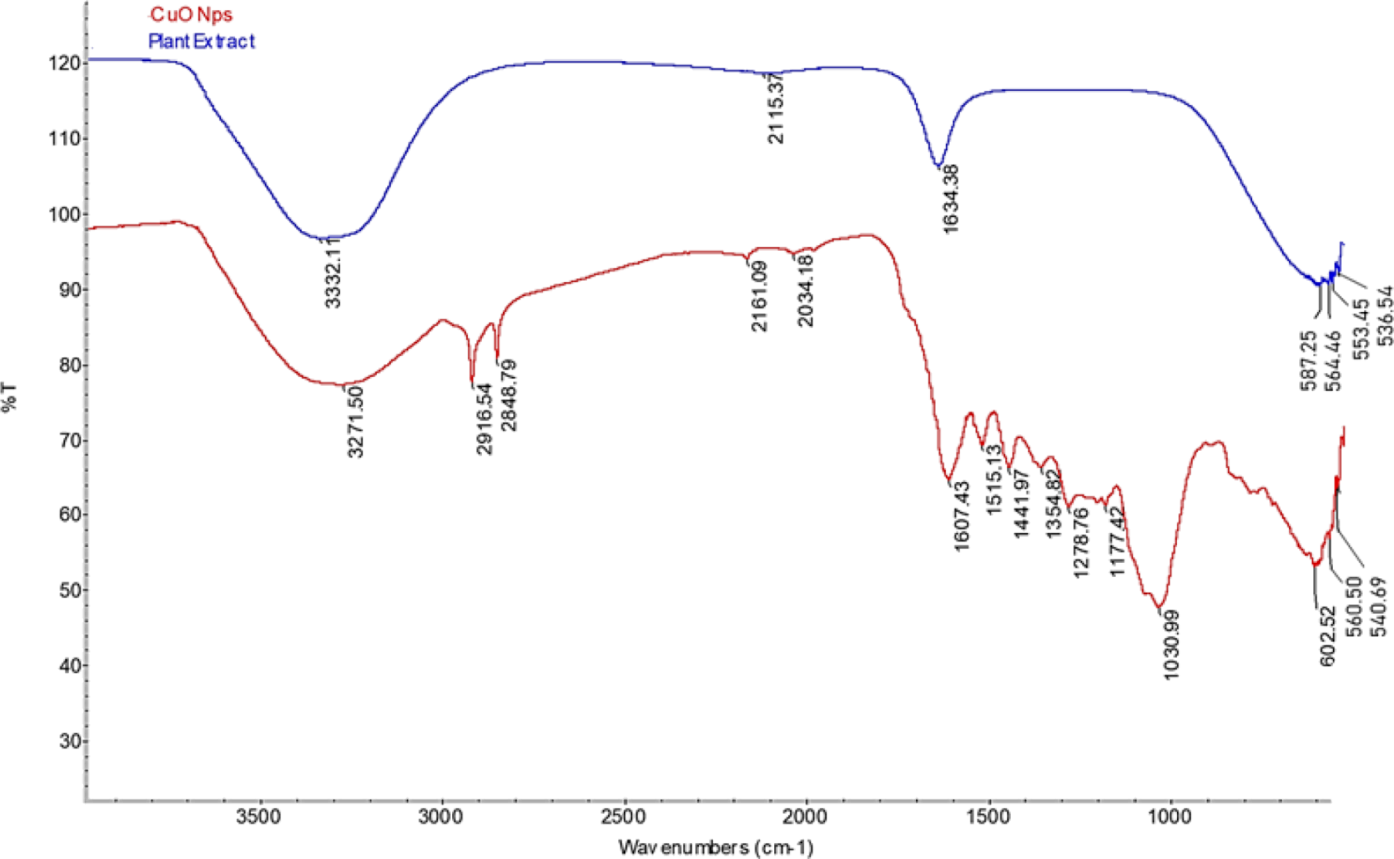
FT-IR spectra of the *A. palmatum* extract (blue) and CuONPs (red).

The structure of CuONPs synthesized via *A. palmatum* leaf extract was investigated by FT-IR in the range of 4000–500 cm^–1^. The differences in peak positions in the IR spectrum of *A. palmatum* leaf extract and CuONPs can be caused by variations in molecular structure and bonding, meaning differentiation of functional groups that can cause shifting in the absorption frequencies and leading to differences in peak positions. The novel peaks obtained by the CuONP IR were monitored at 3271, 2916, 2848, 2161, 2034, 1607, 1515, 1441, 1354, 1278, 1177, 1030, 602, 560, and 540 cm^–1^ (Figure 3). In the spectrum of CuONPs, the broad band at 3271 cm^–1^ can be attributed to −OH stretching, and the sharp peaks at 2916 and 2848 cm^–1^ can be attributed to C–H stretching, while the vibrations around 1607 cm^–1^ correspond to C=O stretching. The peak found at 1515 cm^–1^ is attributed to C=C stretching, whereas the peak at 1441 cm^–1^ is assigned to the presence of the −COO group. The peak at 1354 cm^–1^ may correspond to C–N bending, and the peak at 1278 cm^–1^ is attributed to C–N stretching in amines. The peak at 1177 cm^–1^ is ascribed to the organic functional parts such as amides, ethers, and other aliphatic groups. The strong vibrational peak at 1030 cm^–1^ is contributed to the H–OH bond stretching of alcohols. The peak at 602 cm^–1^ is contributed by C–O bending, while the peaks at 560 and 540 cm^–1^ represent the stretching vibration of the Cu–O bond in monoclinic CuO. Vibrations of CuONP IR indicated the presence of CuONPs by the IR variations of the plant extract and are consistent with the results of other studies in the literature.^27,28^ The new peaks clearly seen in the IR spectrum obtained in the study show that the molecular structure has changed; thus, the functional groups in the structure have been altered and even new functional groups have been added. Therefore, the results of FT-IR analyses indicated the presence and functional groups of the biologically fabricated CuONPs.

### 3.4 SEM Analyses of CuONPs

**Figure 4:**
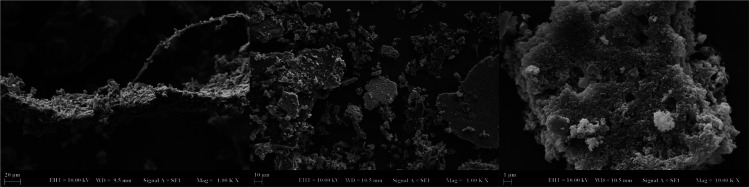
SEM images of CuONPs synthesized via *A. palmatum* leaf extract at various magnifications.

The morphological features and size of the synthesized CuONPs were examined by SEM. The observed images at different magnifications are presented in Figure 4. In the SEM images of CuONPs, it was observed that the nanoparticles were spherical in shape and found in crystal clusters in an agglomerated form. The main reason for the agglomeration is the electrostatic interactions between the spherical crytals, which cause cluster formation. The overlapping of crystal structures is seen in a wide-angle SEM image of 1 μm. The active groups found in the plant extract of *A. palmatum* may also induce agglomeration by the presence of phytochemicals that are responsible for the bioreduction and capping in the reaction environment. SEM images also indicated the particle sizes that ranged from 140 to 225 nm, which are greater in size compared to previous reports in which green and chemical methods were applied.^29,30^ Therefore, identifying the underlying chemical reaction and kinetics for the small size synthesis of CuONPs can be applied by more screening of leaves to investigate the phytochemical components.

### 3.5 DLS Analyses of CuONPs

**Figure 5:**
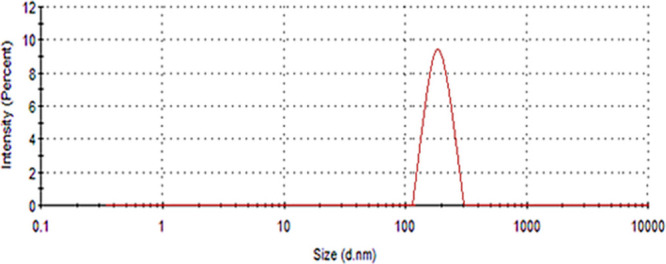
Particle size distribution of CuONPs synthesized via *A. palmatum* leaf extract.

The average particle size and polydispersity index (PDI) value of the synthesized nanoparticles were determined by performing a dynamic light scattering (DLS) analysis of the synthesized CuONPs with a Zetasier Malvern Nano ZS (Figure 5). The average particle size distribution graph of CuONPs was obtained by DLS analysis, and it was found that the spheres had an average diameter of 229 nm. In DLS analysis, the particle size is based on the movement of particles rather than being measured directly of which the result obtained gives the hydrodynamic diameter. In other words, it refers to the size of smooth spheres that have the same velocity as the measured particles. Therefore, in this study, DLS results were compared with other techniques evaluating different physical parameters of nanoparticles. The PDI of CuONPs is 0.184, indicating that the nanoparticles have monodisperse systems. The results of DLS analyses are parallel with previous studies such as DLS analysis of CuONPs synthesized with *Ixora coccinea* leaf extract that had an average nanoparticle size of 167.1 nm and a PDI value of 0.345, which explained that CuONPs were monodisperse.^31^ In general, the PDI ranges of nanoparticles are in the range of 0.01–0.7 values, and particles with a very wide size distribution have PDI values greater than 0.7.^32^ As a result of the study, it was seen that the DLS results we obtained were compatible with those in the literature.

### 3.6 Antibacterial Activity of CuONPs

The antibacterial activity of the biologically fabricated CuONPs was examined (Table 1). While the inhibition zone formed by CuONPs against the Gram-negative *E. coli* strain was 14 mm, the inhibition zone formed against the Gram-positive *S. aureus* strain was measured as 18 mm. The inhibition zone of streptomycin used as a positive control was measured as 20 mm against *E. coli* and 23 mm against *S. aureus*. The antibacterial study results indicated that the synthesized CuONPs showed a strong antibacterial effect, and it was observed that the effect was higher against Gram-positive bacterial strains. In a report examining the antibacterial activity of CuONPs synthesized via tree gum extract, it was observed that the degree of inhibition of bacterial growth depends on the concentration of nanoparticles in the environment. It has been reported that CuONPs act as an excellent antibacterial agent against Gram-positive and Gram-negative bacteria.^33^ The strong antibacterial activity against both types of bacteria as a result of our study can be attributed to the mechanism that starts with the binding of copper ions released by CuONPs with DNA molecules. Copper ions can cross-link within and between nucleic acid strands, leading to disruption of the DNA helix. It is known that copper ions disrupt the structure of bacterial cell membranes, enter the bacterial cells, and damage biochemical processes.^34^

**Table 1:** Diameters of Inhibition Zones of CuONPs and the Controls

	**CuONP**	**negative control (dH_2_O)**	**positive control (streptomycin)**
E. coli	14 mm	-	20 mm
S. aureus	18 mm	-	23 mm

**Figure 6:**
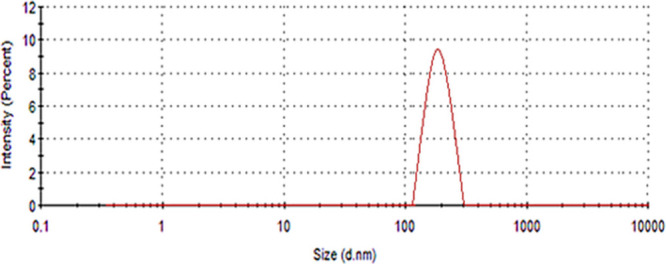
Antibacterial activity of CuONPs fabricated by *A. palmatum* leaf extract according to OD_600_ measurement on the culturing time in the absence and presence of CuONPs using (A) *E. coli* and (B) *S. aureus*.

The quantification of the antibacterial effect of the fabricated CuONPs was evaluated via optical density. The OD_600_ values of the bacterial cultures with or without CuONPs are demonstrated in Figure 6 in which the presence of the copper oxide nanoparticles clearly inhibited the proliferation of both Gram (−) and Gram (+) strains for 24 h. On the contrary, the OD_600_ value of the negative control was increased as time passed, presenting that the employment of the biofabricated CuONPs can cease bacterial growth. The results of the quantitative analysis and disk diffusion assay confirmed the antibacterial activity of the CuONPs biofabricated by using *A. palmatum*. The previous reports on the antibacterial activity of various CuONPs fabricated by using plants implied that the morphology is one of the most important factors, and smaller nanoparticles are preferred.^35^ However, our results showed that CuONPs, which were found to be between 140 and 225 nm in size, exhibited highly effective antibacterial activity. Organic functional groups transferred from the plant and located on the surface of nanoparticles also have an effect on antibacterial performance. The nanoparticle is attached to the bacteria by electrostatic interactions of these functional groups. In this way, it damages the bacterial structure attached to the cell wall, disrupting cell integrity and causing cell death. The fabricated CuONPs’ dispersion capability in a solution is also well as can be seen in the optical analysis. Therefore, the nanoparticles suggested in this report are good candidates of antibacterial agents to their dispersity and morphology.

### 3.7 Antifungal Activity of CuONPs

**Figure 7:**
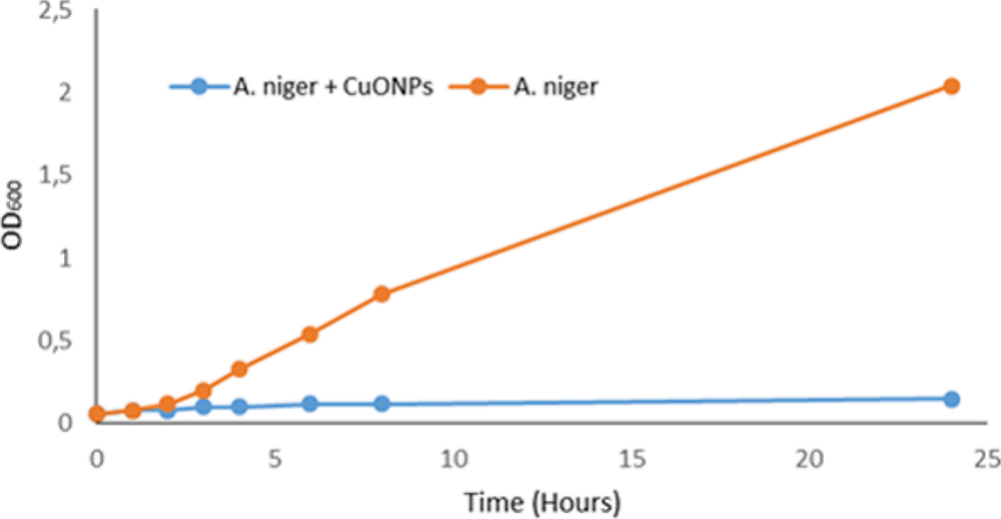
Antifungal activity of CuONPs fabricated by *A. palmatum* leaf extract according to OD_600_ measurement of the culturing time in the absence and presence of CuONPs using *A. niger*.

The antifungal capacity of CuONPs obtained by biological nanofabrication was investigated optically by OD_600_ measurement (Figure 7). The growth inhibition of *A. niger* that may be attributed to the attachment of the CuONPs on the cellular membrane of the fungus and damaging of the cell structure and functions by disrupting the integrity of the membrane can be clearly seen in the graph. The result of the optic measurements demonstrated that the presence of CuONPs supplied a sound antifungal effect on the growth of *A. niger*. Also, the result of the agar diffusion test confirmed the optical density data. The inhibition zone of CuONPs against the *A. niger*stock culture was measured as 24 mm, and the inhibition zone of Amphotericin B, an antifungal antibiotic used as positive control, was 28 mm. The negative control was dH_2_O, which has no clear zone. As a result of the study, it was observed that the synthesized CuONPs had excellent antifungal activity. In a study in which the antifungal activities of CuONPs synthesized from *Brassica oleracea*var. *italic* extract were determined by the disk diffusion method using *Candida albicans* and *A. niger*, it was reported that the size and concentration of CuONPs played an important role in their antimicrobial activity and CuONPs synthesized by green synthesis showed good antifungal activity.^36^

### 3.8 Molecular Docking Analysis of CuONPs

**Figure 8:**
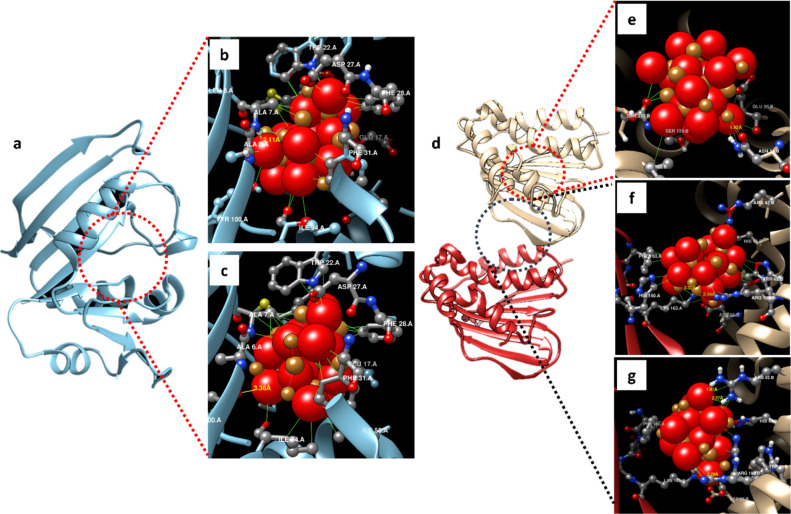
Molecular docking analysis of CuONPs with *E. coli* DHFR and *S. aureus* GyrB. (a) Structure of DHFR (the red dashed line shows the binding site of DHFR); (b) best binding pose calculated by AutoDock Vina and (c) HDOCK. (d) Structure of GyrB (GyrB chain A is the red ribbon, GyrB chain B is the ivory ribbon, the red dashed line shows the region where ATPase activity occurs, and the black dashed line shows the region that best binds with CuONPs). (e) CuONP interactions in the ATPase activity site; (f) pose of the best binding in the junction of subchains of GyrB according to AutoDock Vina and (g) HDOCK (red and gold spheres are CuONP, and interacting residues were depicted as ball and stick).

Local docking analysis was performed via AutoDock Vina (binding site coordinate *x*: −20, *y*: 0, *z*: 10 center with 20 × 20 × 20 Å size). CuONPs have shown −12.562 kcal/mol binding energy in docking with *E. coli* dihydrofolate reductase (DHFR, PDB: 5CC9; Figure 8a). In addition to a 2.08 Å hydrogen bond with Ala7, it has been observed to have 42 interactions with surrounding residues of CuONPs. Most of these interactions occurred with hydrophobic Ala7, Trp22, Phe28, and Phe31 in DHFR, and interaction lengths were between 2.10 and 3.52 Å. In addition, hydophilic interactions were observed with Glu17 and Ser49 and interaction lengths were between 2.57 and 3.73 Å (Figure 8b). On the other hand, CuONPs showed the best docking score with the same binding site in HDOCK blind docking (docking score −188.08). In the binding analysis of HDOCK blind docking, in addition to the 3.36 Å hydrogen bond with Tyr100, interactions of varying lengths between 1.93 and 3.58 Å were observed in Trp22, Phe28, Phe31, Asp27, and Ile94 residues of DHFR (Figure 8c). Both docking algorithms showed comparable interactions between CuONPs and DHFR, suggesting favorable binding between these moieties.

In the molecular docking of *S. aureus* DNA Gyrase B (GyrB; Figure 8d), −8.368 kcal/mol binding energy was obtained by AutoDock Vina in the best binding model (binding site coordinate *x*: 20, *y*: −15, *z*: 5 center with 20 × 20 × 20 Å size). In the best binding model, besides the 1.95 Å hydrogen bond with Asn54, there are many interactions of varying lengths between CuONPs, mostly Ser128, Asn54, and Glu50 (in the range of 1.89–3.54 Å; Figure 8e). On the other hand, when blind docking analysis was performed with HDOCK, interestingly, no binding was observed at this binding site in the best 10 models (Figure 8f, red dashed line). In the HDOCK blind docking analysis, it was found that there could be CuONP interaction in the junction region of the two subchains of the GyrB, in accordance with 9 of 10 models with the best score (Figure 8d, black dashed line). In the best model (docking score −132.26), two hydrogen bonds were observed from the Lys163 residue located in chain A of GyrB (Figure 8f). Since CuONPs interacted with residues from chains A and B, the AutoDock Vina molecular docking was reperformed by giving the interface between two chains as the docking site (*x*: 15, *y*: 0, *z*: 10 center with 20 × 20 × 20 Å size) resulting in −8.797 kcal/mol binding energy. In this interaction, varying lengths of hydrogen bonds were detected with Arg42 and Asp53 in chain B of GyrB. In addition, many interactions were observed from various residues of the two chains (A chain Phe160 and Lys163; B chain Arg42, His46, Asp53, and Agr198) (Figure 8g).

DHFR is an enzyme in the folic acid pathway involved in the reduction of dihydrofolate to tetrahydrofolate, which is crucial in cell proliferation via thymidinylate biosynthesis. It stands out as one of the critical gene products, especially in killing microorganisms that are resistant to antibiotics.^37^ Our findings suggested that the activity of CuONPs on *E. coli* can significantly interfere with DHFR and folic acid metabolism. Another attractive target of antibacterial agents is DNA gyrase. Of the two subunits of DNA gyrase, GyrA mediates DNA double-strand breakage, and the GyrB subunit is responsible for ATPase activity. Antibiotic mechanisms mediated by DNA gyrase inhibition are based on targeting different domains of the two subunits.^38^ In the present study, we found that CuONPs showed high affinity for the interface of the two chains in the GyrB subunit, in addition to significantly targeting the ATPase activity site of the GyrB chain B (Figure 8d–g). Our findings suggested that DHFR and GyrB inhibitions may contribute to CuONP-mediated antibacterial activity in Gram-negative and Gram-positive bacteria, respectively.

### 3.9 Dye Removal Capacity of CuONPs

**Figure 9:**
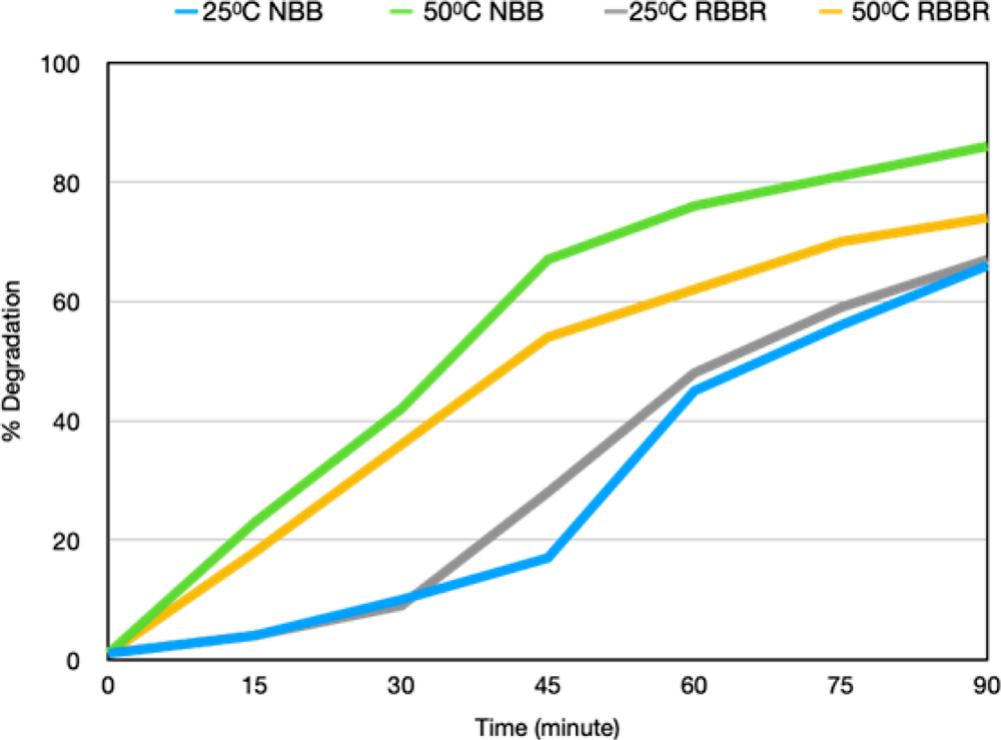
Effect of time and temperature on the dye removal efficiency of the CuONPs.

Two of the most known organopollutants remazol brillant blue R (RBBR) and napthol blue black (NBB) were employed in dye removal applications of the fabricated CuONPs. The graph of %degradation of both dyes against time is represented in Figure 9. Experiments were conducted in two different temperature values, 25 and 50 °C, and an initial dye concentration of 10 mg/L with a constant CuONP amount of 2 mg at pH 5.0. In both cases, the performances of CuONPs were 86 and 74% color removal of NBB and RBBR, respectively, after 90 min of incubation at 50 °C. The figure clearly reveals the percentage of increasing degradation as time progresses. Much of the remazol brillant blue R and napthol blue black were degraded at the end of the period, which is a very short time, resulting in the maximum degradation capacity of the CuONPs. This good performance might depend on the optimum pH conditions because the nanofabricated particles had the maximum efficiency at this pH on both dyes. In addition, the environmental temperature is an important parameter affecting the activity of the CuONPs. The result clearly indicated that the decolorization percentage increases as the temperature increases. Both dyes have an improved degradation rate at 20–40% at 50 °C compared to 25 °C. NBB removal activity of the CuONPs was superior to RBBR, which might be because the structure of NBB is a more suitable substrate for the biologically fabricated CuONPs. Decolorization efficiencies of 67 and 54% were achieved at pH 5.0 after 45 min of incubation, meaning that CuONPs can degrade more than half of both dyes even for such a limited period. There are many reports discussing the metal nanoparticle-supported decolorization of dyes, which reported longer incubation time with parallel results.^39−,41^ It is crucial for the ecosystem that dye-containing effluents be treated before being released into water sources. Therefore, the eco-friendly synthesis and application of CuONPs in industrial dye removal are suggested in light of the results obtained by this study. The synthesized CuONPs are recommended as good candidates as wastewater treatment agents.

## 4 Conclusions

In this study, CuONPs were synthesized by a biological nanofabrication method using *A. palmatum* leaf. A sharp peak at 270 nm was obtained by UV–vis spectroscopy verifying the presence of the CuONPs. In the characterization study performed with FT-IR, the vibrations around 602, 560, and 540 cm^–1^ in the spectrum indicated the presence of CuONPs. The sizes of the synthesized nanoparticles were determined to vary between 140 and 225 nm by SEM analysis, and it was observed that they were almost spherical in shape. The average particle size and PDI values of the nanoparticles were characterized by DLS analysis. It was determined that the nanoparticles synthesized from *A. palmatum* leaf had an average diameter of 229 nm and a PDI value of 0.184, and the PDI values showed that the synthesized nanoparticles were monodisperse systems. The biologically fabricated CuONPs removed napthol blue black and remazol brillant blue R in 90 min at decolorization rates of 86 and 74%, respectively, by demonstrating rapid and good decolorization feature implying the potential to be used against environmental pollution. When the antibacterial activity of CuONPs was determined against Gram (−) *E. coli* and Gram (+) *S. aureus* bacterial strains, it was found that the synthesized nanoparticles had strong antibacterial properties. The antifungal activity of CuONPs against *A. niger* was excellent when compared with that of a commercial antifungal antibiotic. The results of molecular docking analysis have strengthened the phenomenon in which CuONPs may mediate the antibacterial mechanisms through essential bacterial proliferation pathways such as folate metabolism and DNA replication. CuONPs interact with the substrate binding domain of DHFR suggesting an inhibitory effect. In the case of the inhibitory effect of CuONPs on GyrB, it is highly likely that either the ATPase activity site or interchain interface is targeted by CuONPs. This study shows that environmentally friendly CuONPs with antibacterial and antifungal activity can be used in the removal of environmental pollution in addition to different areas. Our research team will continue to concentrate on developing the environmentally friendly synthesis of CuONPs as bactericidal, fungicidal, and decolorative agents.
